# Electrochemical Degradation of Industrial Dyes in Wastewater through the Dissolution of Aluminum Sacrificial Anode of Cu/Al Macro-Corrosion Galvanic Cell

**DOI:** 10.3390/molecules25184108

**Published:** 2020-09-08

**Authors:** Mateusz Łuba, Tomasz Mikołajczyk, Bogusław Pierożyński, Lech Smoczyński, Paweł Wojtacha, Mateusz Kuczyński

**Affiliations:** 1 Department of Chemistry, Faculty of Environmental Management and Agriculture, University of Warmia and Mazury in Olsztyn, Łódzki Square 4, 10-727 Olsztyn, Poland; mateusz.luba@uwm.edu.pl (M.Ł.); lechs@uwm.edu.pl (L.S.); mmateuszkkuczynski@gmail.com (M.K.); 2 Department of Industrial and Food Microbiology, Faculty of Food Science, University of Warmia and Mazury in Olsztyn, Cieszyński Square 1, 10-726 Olsztyn, Poland; pawel.wojtacha@uwm.edu.pl

**Keywords:** electrocoagulation, anodic dissolution, wastewater treatment, surface electrooxidation, galvanic cell, al sacrificial anode

## Abstract

This paper reports on the process of industrial-type wastewater purification carried-out through continuous anodic dissolution of aluminum alloy sacrificial anode for artificially aerated Cu-Al alloy galvanic (macro-corrosion) cells and synthetically prepared wastewater solutions. Electrochemical experiments were performed by means of a laboratory size electrolyzer unit, where the electrocoagulation process along with surface-induced electrooxidation phenomena were examined for wastewater containing Acid Mixture and *Disperse Red 167* dyes. Final reduction of the dyes concentrations came to 32 and 99% for Acid Mixture and *Disperse Red 167*, correspondingly. The above was visualized through the employment of electrochemical (cyclic voltammetry and a.c. impedance spectroscopy techniques) and instrumental spectroscopy analyses.

## 1. Introduction

Water makes an environment for many living organisms, where its quality is crucial for human beings, animals, numerous plants, their habitats, and the ecosystem as a whole. Thus, today maintaining proper purity of water has become of superior importance, not only for local economies, but also from the global point of view. Over the centuries, numerous important methods have been developed for wastewater treatment. These primarily include a combination of the following techniques: physical, mechanical, biological, chemical and others. In general, the process of the purification of wastewater from various pollutants is divided into five successive steps, which comprise: preliminary treatment or pre-treatment of wastewater (physical and mechanical), primary treatment (physico-chemical and chemical), secondary treatment or purification (chemical and biological), tertiary or final treatment (physical and chemical), and final treatment of the formed sludge [1,2]. Nowadays, researchers from around the world strive to improve the commonly used wastewater treatment methods in order to reduce their total costs, optimize efficiency, and to diminish the production of intermediate pollutants. Some of the most promising results were obtained through electrochemical removal of impurities from sewage, where properly designed dc-powered electrochemical systems ensured high efficiency of the wastewater purification treatment [3,4,5,6,7,8,9]. Nevertheless, for electrochemical methods, significant problems could arise during their optimization. An increase in supply’s current-density improves the efficiency of the removal of the contaminants from sewage, but it simultaneously leads to elevation of the process’s total cost. Also, introduction of an insufficient amount of electrical energy into the process would negatively impact the efficiency of wastewater treatment [10]. This problem may be solved by obtaining electricity from renewable energy sources, but the latter (not mentioning significant investment cost) would also require appropriate conditions, e.g., adequate wind strength, sunlight or proper hydroelectricity sources.

On the contrary, the process of municipal (or industrial) wastewater purification could be carried out through electrocoagulation (EC) of pollutants by Al (or Fe) ions that act as destabilizing agents. The EC phenomenon is based on a number of reactions, including: adsorption, electrooxidation and electroflotation processes. The above combines both physical and chemical wastewater treatment methods, but without introduction of chemicals [11]. Apart from a typical dc supply system, aluminum ions may also be generated by galvanic couples, e.g., from aluminum sacrificial anode of aerated, Cu/Al alloy macro-corrosion cell [12,13]. In general, Al alloy is used as a prominent electrode material for sacrificial cathodic protection systems, where the presence of a small amount of other elements (e.g., Hg, In) in bulk Al effectively prevents its surface passivation [14]. On the other hand, Al electrocoagulation is widely envisaged as a potential technique for removing various types of pollutants from wastewaters, e.g., from potable water [15], oil–water emulsions [16,17,18,19], dye-containing solutions [20,21,22,23,24,25], urban, restaurant-based [26,27,28,29,30], and heavy metals containing wastewaters [31,32,33,34].

During the anodic dissolution of Al anode, metal ions that act as a coagulant, become released into the solution at specific conditions and could form a wide range of positively charged species (generated on the cathode), e.g., Al(OH)^2+^, Al(OH)_2_^+^, Al_2_(OH)_2_^4+^, Al_3_(OH)_4_^5+^, Al_6_(OH)_15_^3+^, Al_7_(OH)_17_^4+^, Al_8_(OH)_20_^4+^, Al_13_O_4_(OH)_24_^7+,^ and Al_13_(OH)_34_^5+^ [35,36] that destabilize, flocculate and aggregate particles of the impurities. Destabilization comes from the fact that many pollutants contained in wastewater’s are negatively charged. Thus, the presence of positively charged hydroxyl Al cations leads to neutralization and destabilization of the pollutants, which finally become converted into insoluble forms and precipitate on the tank’s bottom. The above results from the fact that the high surface-area structured Al hydroxyl complexes strongly facilitate the formation of the pollutants/Al_x_(OH)_y_^n+^ agglomerates. Then, such formed agglomerates would easily be removed by filtration, because of the relatively large density of in situ generated flocs [37,38]. 

A key advantage of the macro corrosion-cell driven process over a typical dc-powered Al electrocoagulation is that the former is purely based on the theory of a galvanic cell’s operation, where dissolution rate of the anode is controlled by an open-circuit voltage of the cell’s constituents, as well as the presence of a depolarizing agent-dissolved oxygen molecules in most cases. Here, there is no necessity for expensive, dc rectifier infrastructure and control systems, and thus the consumption of electrical energy is radically limited. Based on the galvanic series theory, in the case of a Cu/Al alloy cell, an Al-based electrode makes an anode, whereas Cu plates are located on both sides of the anode constitute cathodes. The Cu/Al galvanic cell being in operation could drive the following anodic and cathodic reactions (Equations (1)–(5)):

Anodic reactions:

(1) $$Al_{\left(s\right)}\to Al^{3+}+3e^{-}$$

(2) $$2H_{2}O_{\left(l\right)}\to O_{2}↑+4H^{+}+4e^{-}$$

Cathodic reactions:

(3) $$2H_{2}O+2e^{-}\to H_{2}↑+2OH^{-}$$

(4) $$O_{2}+2H_{2}O+4e^{-}\to 4OH^{-}$$

(5) $$Al^{3+}+3OH^{-}\to Al{\left(OH\right)}_{3\;}↓$$

**Figure 1:**
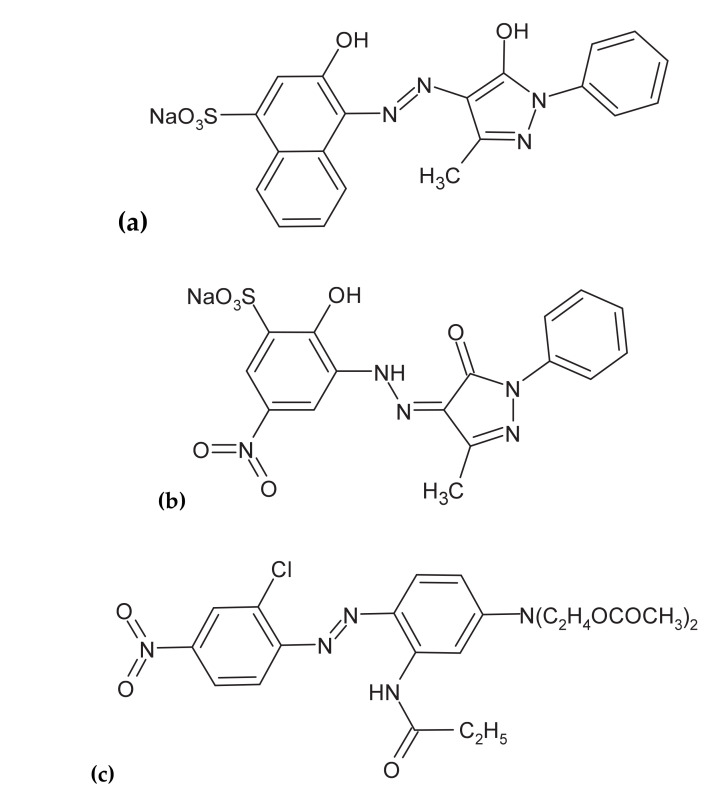
Chemical structures of Acid Mixture: (**a**) *Acid Violet 90* and (**b**) *Acid Red 357*; and (**c**) *Disperse Red 167* molecules.

Thus, Al particles released into the wastewater solution cause an increase in the Al ions concentration. They are immediately hydrolyzed to polymeric aluminum hydroxides that are considered perfect coagulation agents for various pollutant molecules. It has previously been confirmed that these compounds have a strong affinity to dispersed molecules, which finally form large agglomerates at the bottom of the sedimentation tank and could eventually be siphoned off through the filtration process [39,40]. On the other hand, the removal of the agglomerates is facilitated by the hydrogen and oxygen gas bubbles formed on the surface of both cathode and anode, see Equations (2) and (3). They are responsible for electroflotation of the formed insoluble pollutants to the surface of the tank. Such-generated contaminants layer on the surface of wastewater could then be mechanically removed. In this study, the galvanic cell in the form of the sacrificial Al alloy anode and Cu cathodes was used to examine the efficiency of electrochemical purification (envisaged as a combined effect of electrochemical coagulation and surface-electrooxidation processes) of artificially-prepared industrial wastewater containing small amounts of two technologically important dyes, namely Acid Mixture (*Acid Violet* 90 and *Acid Red 357*) or *Disperse Red 167* (see their chemical structures in Figure 1a–c below).

## 2. Results and Discussion

### 2.1. Characterization of Macro-Corrosion, Galvanic Cell’s Operation

**Figure 2:**
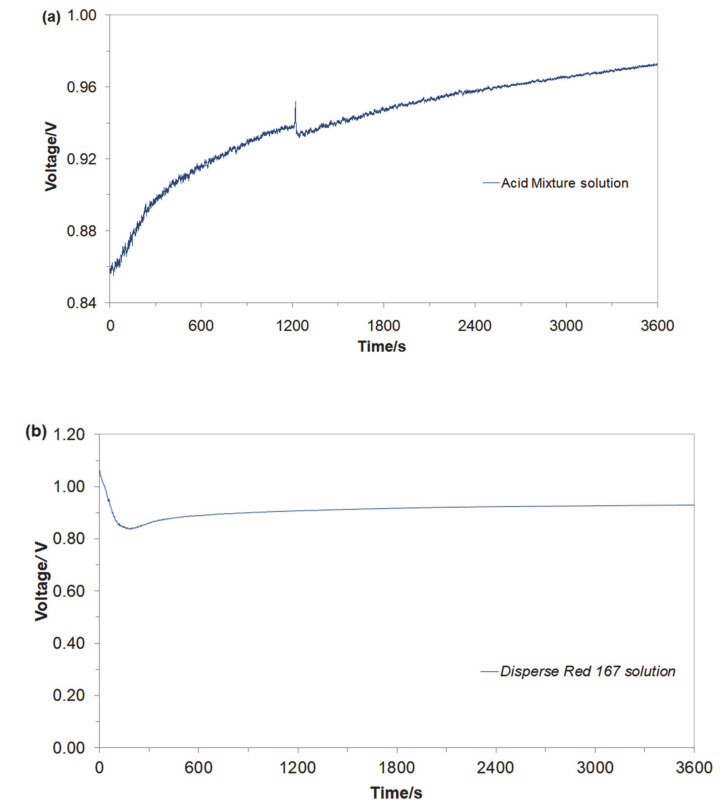
Recorded values of open-circuit voltages for Cu/Al alloy galvanic cell, derived for freshly-prepared, aerated Na_2_SO_4_-based solutions, in the presence of (**a**) Acid Mixture and (**b**) *Disperse Red 167* dyes, both at the concentration of 50 mg dm^−3^.

Initially, an open-circuit voltage (*ocv*) parameter for the Cu/Al alloy galvanic cell was examined in aerated, Na_2_SO_4-_supporting electrolyte (pH = 3 and 4.5 for the Acid Mixture and *Disperse Red 167*-based solutions, respectively; κ = 10.0 mS cm^−1^: 0.054 M Na_2_SO_4_ and t = 20 °C). Hence, after about an hour, the Cu/Al alloy galvanic cell for the Acid Mixture and *Disperse Red 167* dye containing solutions exhibited the following steady values of open-circuit voltages: 0.97 V and 0.92 V, correspondingly (see Figure 2a,b).

**Figure 3:**
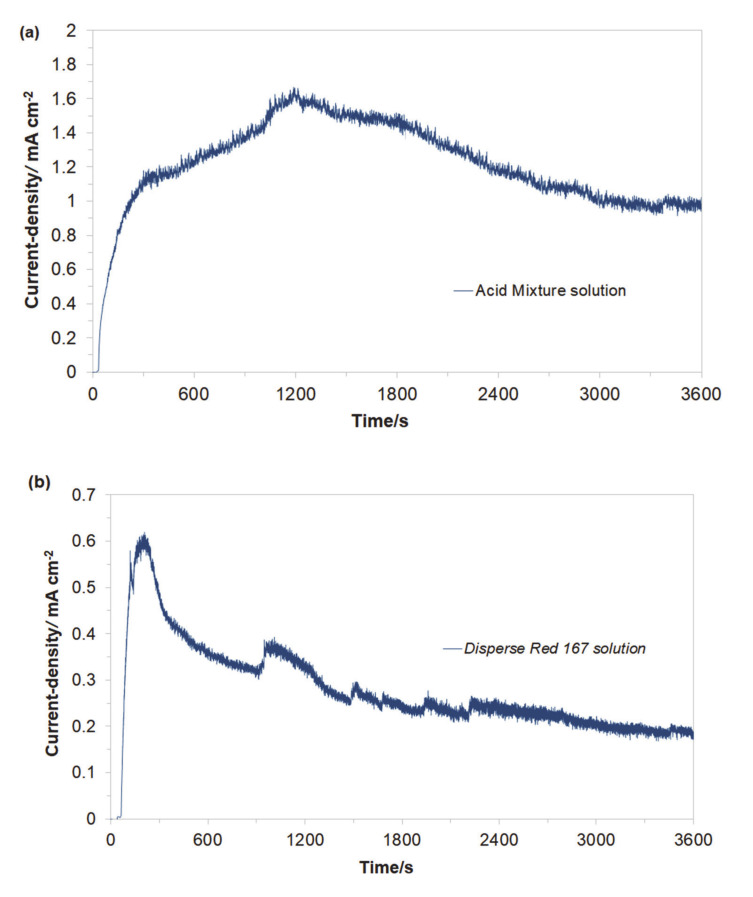
Recorded galvanic couple current-densities for macro-corrosion Cu/Al alloy galvanic cell in function of exposure time, derived for freshly-prepared, aerated Na_2_SO_4_-based solutions, containing (**a**) Acid Mixture and (**b**) *Disperse Red 167* dyes, both at the concentration of 50 mg dm^−3^.

Then, the electrochemical performance for the galvanic coupling in the respective two solutions is shown in Figure 3a,b. Hence, the Cu/Al alloy galvanic cell exhibited galvanic current values (*I*_gc_) of about 44 mA (1.16 mA cm^−2^) and 7 mA (0.18 mA cm^−2^) after 1 h of continuous exposure for the Acid Mixture and *Disperse Red 167* dye-based solutions, respectively. In both cases, an anodic process corresponds to the dissolution of the Al sacrificial anode, Equation (1). A radical reduction of current intensity for the Cu/Al alloy galvanic cell, recorded in the presence of the *Disperse Red 167* dye (Figure 2b), is most likely associated with significant enhancement of its surface adsorption properties (as compared to those exhibited by the Acid Mixture dye). Furthermore, it has to be stated that employment of pure aluminum anodes results in radically inhibited anode oxidation, as elemental Al (unlike some of its alloys) is highly-susceptible to the surface formation of a thick, electrically insulating and exceptionally corrosion-resistant oxide layer (see e.g., recent work by Pierozynski and Piotrowska in Ref. [12]).

Hence, Al^3+^ cations released during anodic dissolution reaction act as the so-called “coagulation agent”. The process of anodic dissolution is accompanied by hydrogen evolution reaction, Equation (3), or predominantly by oxygen reduction reaction, Equation (4), proceeding on the surface of Cu cathodes. The above leads to a gradual increase of solution’s pH and finally results in the extensive formation of aluminum hydroxide species, where: Al^3+^ and Al(OH)_2_^+^ are specific for pH values of 2–3, and a polymeric species: Al_13_O_4_(OH)_24_^7+^ is being formed over the pH range: 4–9, which then becomes precipitated as Al(OH)_3_ solid particles [40].

### 2.2. Electrochemical Characterization of Acid Mixture and Disperse Red 167 Dyes Oxidation Reactions

**Figure 4:**
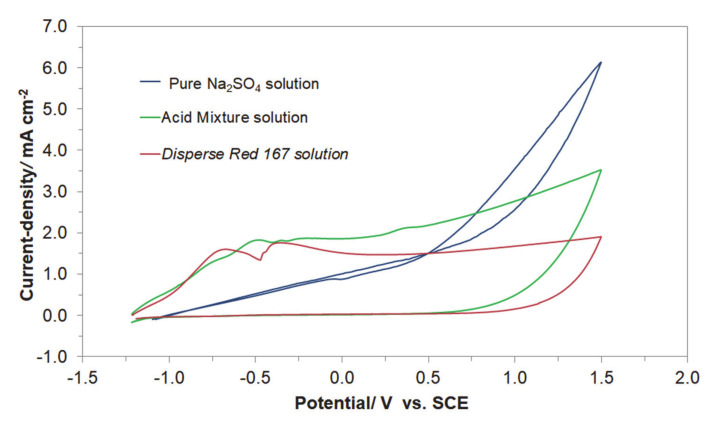
Cyclic voltammograms recorded for Al alloy anode in 0.054 M Na_2_SO_4_ electrolyte and in the presence of Acid Mixture, and *Disperse Red 167* dyes, both at the concentration of 50 mg dm^−3^, at a sweep-rate of 50 mV s^−1^.

Figure 4 presents the cyclic voltammetry behaviour of the Acid Mixture and *Disperse Red 167* dyes, carried out at room temperature on Al alloy working electrode (for the Cu/Al alloy galvanic cell) in 0.054 M Na_2_SO_4_ supporting electrolyte, at a sweep-rate of 50 mV s^−1^. The CV curve recorded in unmodified Na_2_SO_4_ presents Al alloy anodizing behaviour with current-density linearly increasing with potential (significantly different from that recorded on high purity Al electrodes, with extended formation of surface oxide layer, see e.g., details in Ref. [14]), where a radical rise in the current-density beyond the potential of ca. 0.70 V/SCE implies onset of oxygen evolution reaction (OER) on the anode surface.

On the contrary, the presence of the Acid Mixture and *Disperse Red 167* dyes in the supporting solution results in the voltammetric profiles resembling those of surface-electrosorbed (or partly blocked) electrode. Here, a series of clearly pronounced oxidation peaks could be observed, centered at the following potential values: −0.75, −0.48, −0.24, and 0.37 V/SCE for the Acid Mixture dye and at −0.67 and −0.37 V for the *Disperse Red 167* pigment. These peaks are most likely associated with surface oxidation processes involving adsorbed molecules of the examined dyes. Also, as the surface of Al alloy electrode is considerably blocked by the dyes molecules, further anode surface oxidation and eventually the process of the OER in the presence of dyes become significantly inhibited (see Figure 4).

### 2.3. Electrochemical Impedance Characterization 

**Figure 5:**
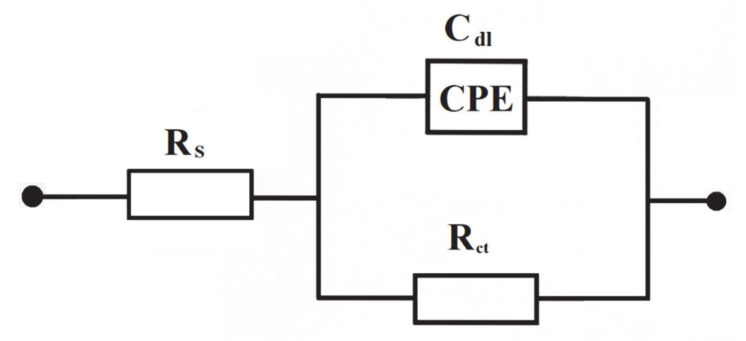
Equivalent circuit model used in a.c. impedance data modeling for the process of electrooxidation of azo dyes on the surface of Al alloy anode in Na_2_SO_4_ supporting solution. The circuit contains a double-layer capacitance, *C*_dl_ (as CPE: Constant Phase Element for distributed capacitance) and charge-transfer resistance, *R*_ct_, while *R*_s_ is solution resistance.

**Figure 6:**
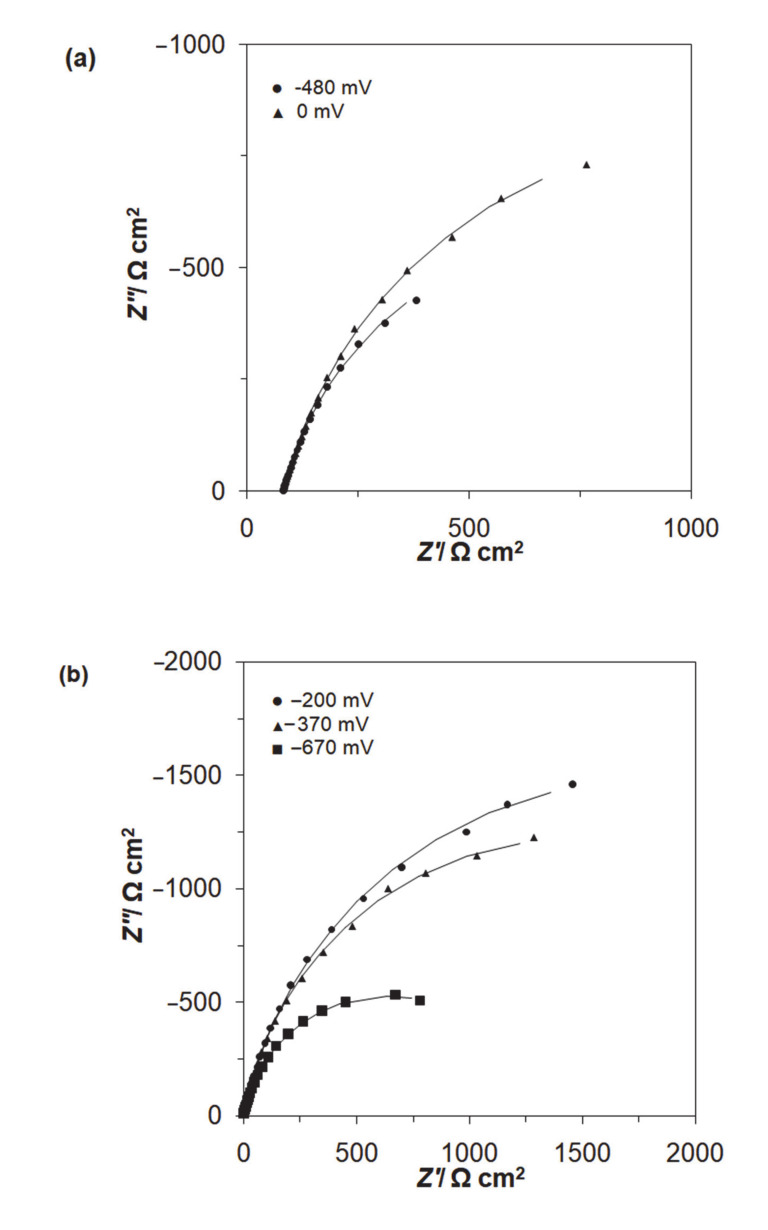
Complex plane Nyquist impedance plots for Al alloy electrode in contact with (**a**) 0.054 M Na_2_SO_4_-based Acid Mixture solution, (**b**) 0.054 M Na_2_SO_4_-based *Disperse Red 167* solution, both dyes at the concentration of 50 mg dm^−3^, recorded for the stated potential values vs. SCE. The solid lines correspond to the representation of the data according to the equivalent circuit shown in Figure 5.

A.c. impedance characterization of Al alloy surface reactivity of Acid Mixture and *Disperse Red 167* dyes in 0.054 M Na_2_SO_4_ is shown in Figure 5 and Figure 6, and Table 1 below. The impedance experiments were carried-out at the selected electrode potentials, in reference to the CV-observed oxidation peaks in Figure 4 above. Hence, all the recorded impedance spectra exhibited single, somewhat “depressed” semicircles in relation to a single-step charge-transfer reaction, in the explored frequency range (see Nyquist impedance plots shown in Figure 6a,b). Thus, for the peak current-density potentials of −670 and −370 mV vs. SCE (see the CV profile for the *Disperse Red 167* dye in Figure 4), the recorded Faradaic reaction resistance (*R*_ct_) parameter values came to 1263 and 2866 Ω cm^2^. Then, at the potential of −200 mV (just beyond the second oxidation peak), the *R*_ct_ parameter radically increased to reach 3565 Ω cm^2^. On the other hand, for the Acid Mixture dye, the recorded charge-transfer resistance parameter reached 1457 Ω cm^2^ at the peak current-density potential of −480 mV and 2009 Ω cm^2^ over voltammetric plateau region (at 0 mV). As the recorded resistance values over the characteristic voltammetric peaks tend to be radically smaller than those derived for the potentials positive to these anodic features (this region corresponds to successive surface electrooxidation of the Al alloy), they are claimed to be associated with additional surface oxidation process(es)—namely with electrodegradation of the surface-adsorbed dye molecules (also, compare with the impedance behaviour recorded for high passivity and porous aluminum alloys in Refs. [41,42,43]).

Then, the double-layer capacitance (*C*_dl_) parameter, recorded based on a constant phase element (CPE)-modified Randles equivalent circuit presented in Figure 5, fluctuated between 78 and 171 μF cm^−2^ (see details in Table 1). The CPE element is included in the equivalent circuit in order to account for the so-called capacitance dispersion [44] effect, represented by distorted semicircles in the Nyquist impedance spectra (Figure 6). Taking into account a commonly used value of 20 μF cm^−2^ in the literature for smooth and homogeneous surfaces [45], the surface roughness factor for the Al alloy anode could be estimated at about 3.9–8.5×.

### 2.4. UV-VIS Spectrophotometry Analysis

In this study, the process of Al alloy surface electrodegradation of the examined azo dyes upon the operation of the Cu/Al alloy galvanic cell was monitored by means of UV-VIS spectrophotometry technique, where the process’s optimization was carried-out by recording absorption spectra over 200–999 nm wavelength range.

It is well-known [46,47,48] that the oxidation process of the azo dyes typically begins with a break of the azo bond (a decolourization stage). It is then followed by the degradation of benzene ring in the dye molecule to finally undergo complete or partial mineralization process. These effects could be visualized by absorbance changes within the UV-VIS spectrum for the selected wavelength ranges, namely: 350–600, 265–350 and 200–265 nm, correspondingly [46,47,48,49,50,51].

**Figure 7:**
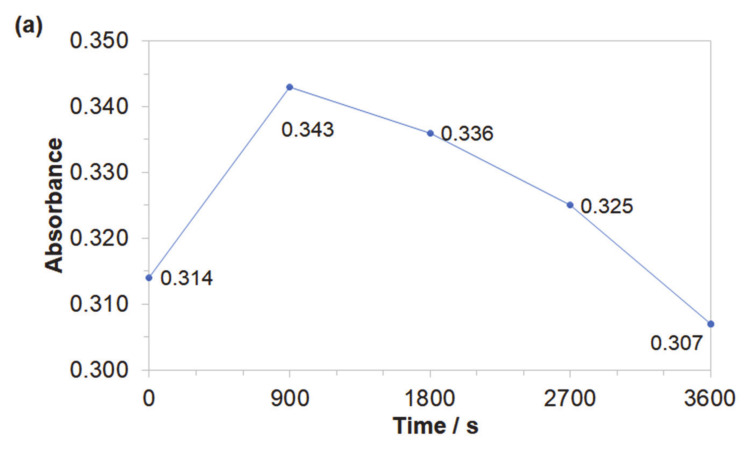

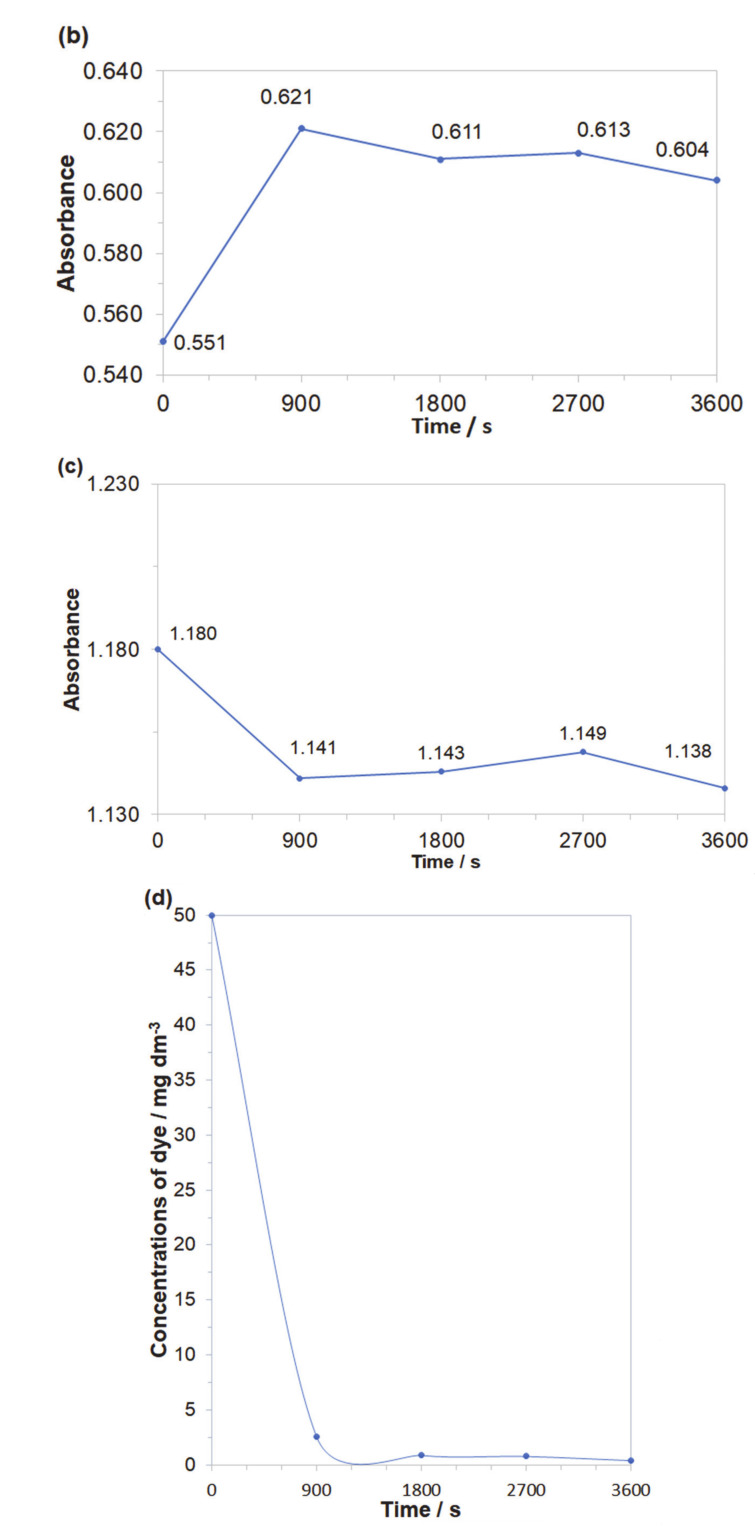
Representation of the three segments of dye electrooxidation process on the surface of Al alloy anode in Na_2_SO_4_-based *Disperse Red 167* solution (at the concentration of 50 mg dm^−3^): (**a**) Evolution of decolourization process recorded for the wavelength of 470 nm; (**b**) Progress of aromatic ring degradation for the wavelength of 280 nm; (**c**) Evolution of mineralization process recorded for the wavelength of 240 nm; (**d**) Quantitative dye assay after sedimentation process for the wavelength of 470 nm.

**Figure 8:**
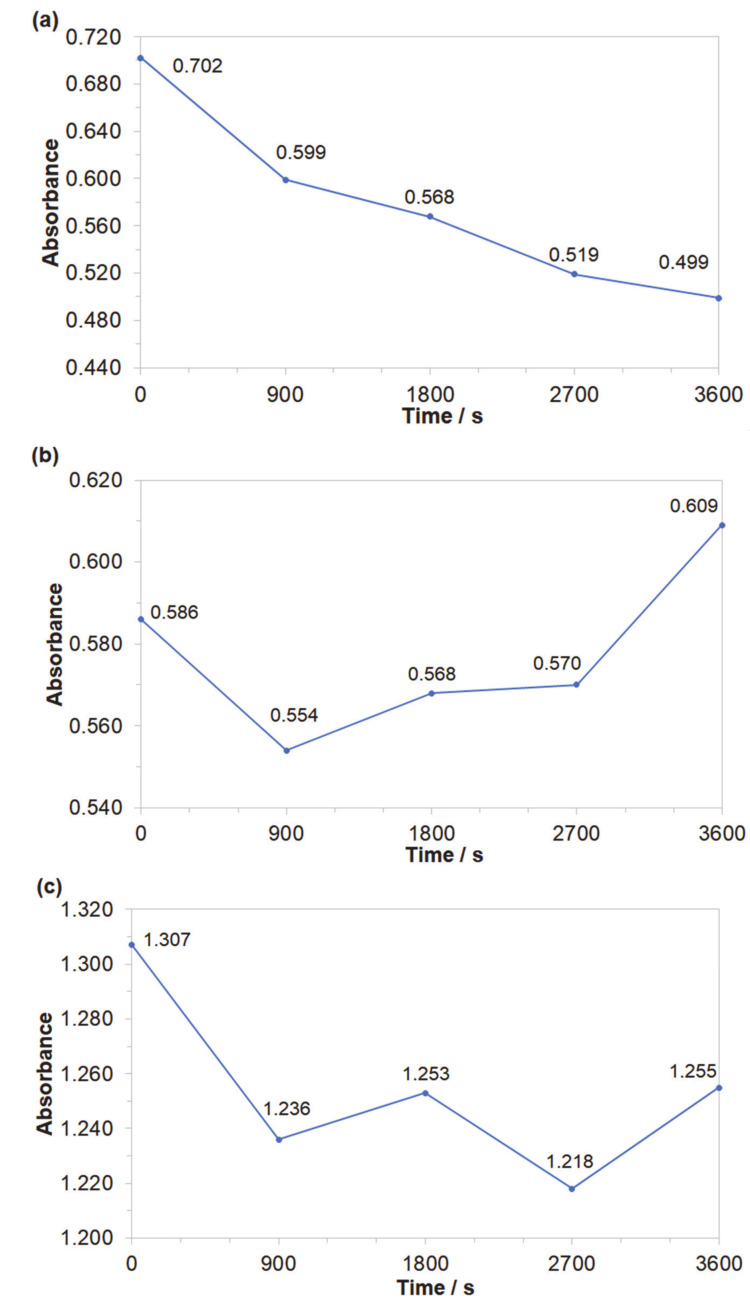

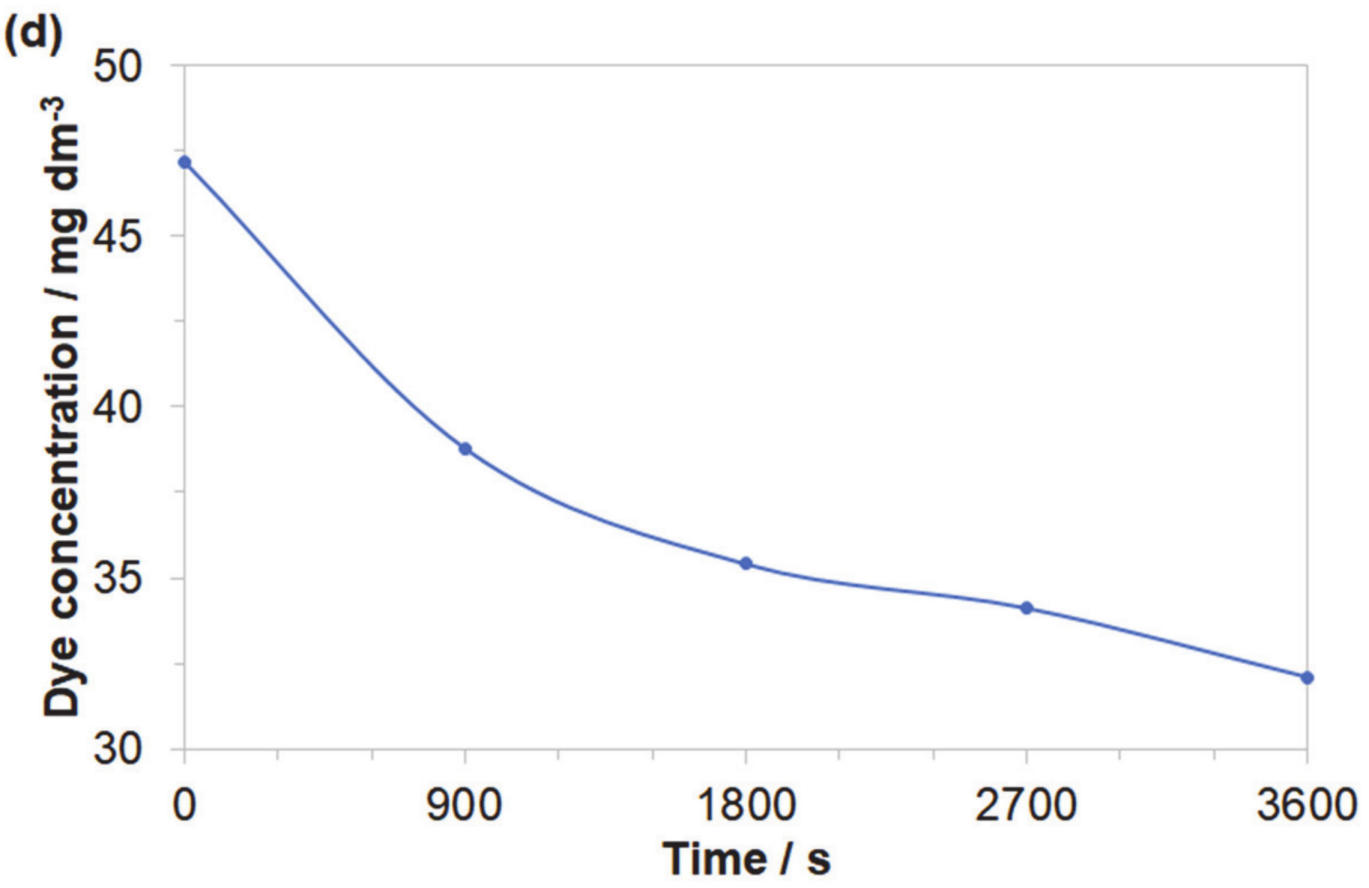
Representation of the three segments of dye electrooxidation process on the surface of Al alloy anode in Na_2_SO_4_-based Acid Mixture solution (at the concentration of 50 mg dm^−3^): (**a**) Evolution of decolourization process recorded for the wavelength of 500 nm; (**b**) Progress of aromatic ring degradation recorded for the wavelength of 280 nm; (**c**) Evolution of mineralization process recorded for the wavelength of 240 nm; (**d**) Quantitative dye assay after sedimentation process for the wavelength of 500 nm.

Obtained results indicated that both azo dyes underwent degradation during the electrolysis process. The effect of an initial decomposition stage is illustrated in Figure 7a and Figure 8a for wavelengths of 470 and 500 nm, corresponding to the *Disperse Red 167* and Acid Mixture. Here, the recorded diminishing absorbance is directly linked to the concentration reduction of both pigments. During the decolourization stage, the azo bond (–N=N–), which is a chromogenic group, becomes an acceptor for electrons released during the anodic oxidation process. Thus, the dye molecule breaks down into aromatic/phenol and aromatic amine derivatives, which are structurally similar to 3-Amino-2-hydroxy-5-nitrobenzenesulfonic acid or 2-chloro-4-nitrobenzenamine [46,47,48]. The increase in the absorbance recorded for the *Disperse Red 167* during the first 900 s of the experiment was most likely connected with partial multimerization of the electrolysis products or else with the process of electrochemical coagulation [52,53,54,55]. With respect to the aromatic ring degradation stage, over the initial 900 s of the *Disperse Red 167* dye electrolysis, the absorbance (Figure 7b) radically increased from 0.551 to 0.621, which is most likely associated with the concentration rise of the aromatic type of substances. Then, a sluggish reduction of the absorbance parameter over the next 45 min. corresponds to a limited efficiency (see Figure 7a again) of the initial dye degradation stage. As the initial decolourization process for the Acid Mixture pigment was significantly more efficient than that of the *Disperse Red 167*, the reaction effectiveness for the second electrodegradation stage was very high up to 2700 s, when it radically dropped to reach the absorbance value of 0.609 after 1 h of the electrolysis (Figure 8b). It is strongly believed that the latter effect is a consequence of accumulation of the reaction intermediates from the first electrodegradation stage in bulk of the analyzed solution. Last, pigments electrodegradation process (Figure 7c and Figure 8c) is related to mineralization, where a vast number of relatively simple (e.g., maleic, formic and acetic acid, aldehydes, ammonia, nitrogen oxides and finally carbon dioxide molecule) or more complex chemical compounds could be formed, such as: anti-pentanoic acid and others [48,49,50,51]. It has to be stated that for the *Disperse Red 167,* its removal involved a combination of two steps, namely: surface electrooxidation and electrocoagulation processes, yielding 98 and 99% of the dye removal after 900 and 3600 s, respectively (Figure 7d). On the contrary, for the Acid Mixture dye, a primary electrooxidation process was observed, leading to 22% and 32% removal after 900 and 3600 s, correspondingly (Figure 8d). 

## 3. Materials and Methods 

### 3.1. Macro-Galvanic Cell’s Construction and Electrolysis Process

**Figure 9:**
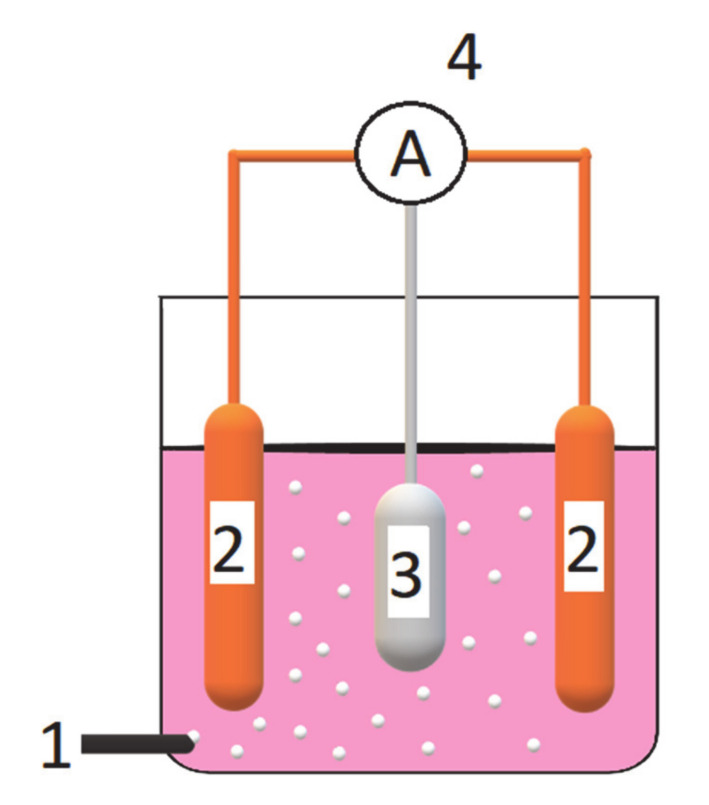
Schematic illustration of galvanic cell-based wastewater electrolysis system, where: 1—is an aeration system (oxygen was delivered from compressed 200 bar cylinder); 2—are copper cathodes; 3—is an Al alloy sacrificial anode; and 4—is an ammeter and electrical connections.

The electrocoagulation unit consisted of a 300 cm^−3^ glass-made electrochemical reactor (see its schematic representation in Figure 9 below) with the square-shaped, single sacrificial anode in the center [Al alloy (surface area: S_Al_ ~ 38 cm^2^; cylindrical shape with a diameter of ϕ = 4 cm and thickness: d = 1 cm): 3.0–5.5 wt.% Zn + 0.016–0.040 wt.% In (MAKROMOR, Gdansk, Poland)] and two copper cathodes (S_Cu_ ~ 84 cm^2^; plates: 4 × 5 × 0.1 cm) located at two opposite sides of the electrolysis cell. The anode-to-cathode distance was kept constant at about 20 mm.

Before being positioned in the cell, the sacrificial anode was first polished with emery paper down to 2500 grade; then, it was activated for 5 min in 5% HCl solution before being rinsed with ultra-pure water (18.2 MΩ Millipore Q3 UV water purification system manufactured by Millipore/Merck was used) and finally de-greased in pure ethanol. On the other hand, Cu cathodes were activated in 5% HCl, thoroughly rinsed with ultra-pure water, and finally de-greased in ethanol. The electrolysis cell was operated at room temperature with additional aeration (by slow purging of compressed oxygen in order to reach the value of ca. 10.5 ppm of dissolved oxygen). Open-circuit voltages were always measured prior to and after completion of the electrochemical tests.

### 3.2. Solutions and Chemical Reagents

The effectiveness of electrocoagulation and surface electrooxidation processes was examined for two synthetic dyes, namely: Acid Mixture (*Acid Violet 90* and *Acid Red 357*) and *Disperse Red 167* (Boruta-Zachem SA, p.a.; Bydgoszcz, Poland) in sodium sulphate supporting solution. These were prepared by dissolving 50 mg dm^−3^ of the respective dyes in 0.054 M Na_2_SO_4_ (Polish Chemical Compounds, p.a.) solution. In order to avoid spontaneous chemical coagulation, the pH for both working solutions was adjusted to the set values of 3.0 and 4.5 for the Acid Mixture and *Disperse Red 167* pigments, respectively. Then, to maintain the correct pH of the working media, dilute solutions of 0.1 M sodium hydroxide and 0.1 M sulphuric acid were used. HCl was not employed simply to avoid possible chlorination effects of aromatic pollutants that could normally be present in industrial wastewaters. On the other hand, Na_2_SO_4_ salt base was used to provide the necessary wastewater conductivity, required for the galvanic cell’s operation.

### 3.3. Experimental Methodology

All galvanic current measurements were conducted by means of the Solartron 12,608 W Full Electrochemical System (ZRA mode). Otherwise, electrolyte conductivity and pH evaluations were carried-out with HI 9835 and HI 2002-01 meters from Hanna Instruments (Smithfield, RI, USA), correspondingly. For instrumental electrochemical measurements (a.c. impedance spectroscopy and cyclic voltammetry tests), the Solartron 12,608 W System was also employed. All electrochemical tests were conducted at room temperature (298 ± 1 K). The electrochemical impedance spectroscopy measurements were carried out at an a.c. signal of 5 mV and the frequency was swept between 1.0 × 10^5^ and 0.5 × 10^−1^ Hz, whereas cyclic voltammetry (CV) experiments were performed at a sweep-rate of 50 mV s^−1^. *ZPlot 2.9* and *Corrware 2.9* software packages (Windows, Scribner Associates, Inc.) were employed to control the instruments. Data analysis was carried out by means of *ZView 2.9* (*Corrview 2.9*) software packs, where the impedance spectra were fitted with a complex, non-linear, least-squares immittance fitting program, *LEVM 6*, written by J.R. Macdonald [56]. Before the cyclic voltammetry and the impedance measurements, working electrodes were cathodically treated at −100 mA for 600 s in pure 0.054 M Na_2_SO_4_ solution in order to reduce the surfaces oxides formed during previous experiments. The CV experiments were conducted over the potential range: −1.2 V to 1.5 V vs. SCE, commencing the sweep from the most negative electrode potential.

In order to view the progress of electrochemical degradation of dyes over time, the solution samples were collected after 900, 1800, 2700, and 3600 s of the Cu/Al alloy cell’s continuous operation (in reference to Figure 3) for both dye-based wastewater solutions. Then, efficiency evaluation of the carried-out wastewater purification treatments was based on the application of UV-VIS spectroscopy analysis. The UV-VIS absorption spectra for the *Disperse Red 167* and Acid Mixture azo dyes were recorded by means of a spectrophotometric plate reader EPOCH 2 (BIOTEK, Winooski, VT, USA) on polystyrene plates (UV-Star^®^ Microplates, Greiner Bio One, Frickenhausen, Germany). Before conducting the measurements, all samples were homogenized by means of a vortex device (IKA Verke, Staufen im Breisgau, Germany), in order to keep all particles in bulk solution. The absorption spectra for all the collected samples were recorded for wavelengths between 230 and 999 nm. A degree of specific dye degradation was then assessed at all electrolysis times, namely: 0, 900, 1800, 2700, and 3600 s. Then, quantitative measurements were carried out after allowing the specimens to sediment (5 min) on the vials’ bottom. These measurements were performed at wavelengths of 500 and 470 nm for Acid Mixture and *Disperse Red 167*, respectively. Such-obtained results were then analyzed with Graph Pad Prism 6 software (Graph Pad, San Diego, CA, USA).

## 4. Conclusions

Efficient purification of azo dye-based wastewater could successfully be performed through a combination of electrocoagulation and surface electrooxidation processes through the employment of Cu/Al alloy macro-corrosion galvanic couple. A major advantage of the corrosion-cell-driven (over the direct current-powered) electrocoagulation process is that the former one is much more cost-effective, especially with respect to lack of expensive rectifier infrastructure. Also, aluminum alloy sacrificial anode is not prone to surface passivation, which makes it superior in this application to that of pure Al. In addition, conducted spectrophotometric UV-VIS instrumental analysis implied that both *Disperse Red 167*, as well as Acid Mixture dyes, underwent significant destruction of their chemical structures, leading to the formation of relatively simple electrodegradation products. Involvement of surface (Al alloy) electrooxidation reactions along with the formation of surface-electrosorbed intermediates was strongly supported by the recorded a.c. impedance and cyclic voltammetry results. Combined electrodegradation and electrocoagulation processes allowed to obtain final percentage removal at levels of 32% (Acid Mixture) and 99% for *Disperse Red 167* dye. 

Further work along with optimization of major parameters of the process is necessary in order to assess the suitability of this method for a technical scale and continuous, dye-based and environmentally-friendly wastewater purification system.

## 5. Patents

Pierożyński, B.; Smoczyński, L. Electrocoagulator for wastewater treatment, Patent of the Republic of Poland, PAT.227874, granted on 5 September 2017.

## Figures and Tables

**Table 1:** Parameters for the process of azo dyes electrooxidation (at a total concentration of 50 mg dm^−3^) on Al-alloy sacrificial anode in contact with 0.054 M Na_2_SO_4_, achieved by fitting the equivalent circuit model shown in Figure 5 to the experimentally-obtained impedance data [dimensionless φ parameter, which determines the constant phase angle in the complex-plane plot (0 ≤ φ ≤ 1) of the CPE circuit, varied between 0.84 and 0.89].

*E*/mV	*R*_ct_/Ω cm^2^	*C*_dl_/µF cm^−2^
*Disperse Red 167*		
−670	1263 ± 21	108 ± 2
−370	2866 ± 62	84 ± 1
−200	3565 ± 30	78 ± 1
Acid Mixture		
−480	1457 ± 24	171 ± 3
0	2009 ± 80	129 ± 3
